# Retraction: Exploring Occupational Health Challenges in Pathology: A Cross-Sectional Survey of Indian Pathologists

**DOI:** 10.7759/cureus.r191

**Published:** 2025-08-26

**Authors:** Rahul Kanungo, Anjali Sharma, Sajal Pagi, Kanika Sachar

**Affiliations:** 1 Pathology, BLDE (Deemed to be University) Shri B.M. Patil Medical College, Hospital, and Research Centre, Vijayapura, IND

The Editors-in-Chief are retracting this article due to multiple data discrepancies throughout the article. The authors did not respond to multiple requests for clarification. As a result, the journal no longer has confidence in the results of this study.

